# Opiate Dependence as an Independent and Interactive Risk Factor for Arterial Stiffness and Cardiovascular Ageing - A Longitudinal Study in Females

**DOI:** 10.4021/jocmr1496w

**Published:** 2013-08-05

**Authors:** Albert Stuart Reece, Gary Kenneth Hulse

**Affiliations:** a School of Psychiatry and Clinical Neurosciences, University of Western Australia, Crawley WA 6009, Australia

**Keywords:** Arterial stiffness, Heroin, Opiate dependence, Vascular ageing, Human ageing

## Abstract

**Background:**

Despite intriguing observational cross-sectional data there are no longitudinal studies of opiate related arterial disease. As opiates act via P16INK4A/CDKN2A, and vascular ageing has been thought to be a surrogate for organismal ageing, the subject has far-reaching implications.

**Methods:**

Pulse Wave Analysis (PWA) by radial arterial tonometry (SphygmoCor) was performed on control and opiate dependent patients.

**Results:**

A total of 37 controls were compared with 93 opiate dependents. They were studied on 117 and 275 occasions respectively up to 1,797 days. The mean (± S.E.M.) ages were 38.72 ± 2.64 and 33.78 ± 0.90 years (P = 0.0260), 91.4% and 10.8% smoked (P < 0.0001). Body mass index rose more in controls (P = 0.0185) and in interaction with time (P = 0.0025). When controlled for time and BMI, opiate dependency status was shown to be associated with vascular age and central arterial stiffness and pressure indices (all P < 0.05). When repeated measures multiple regression was performed on all traditional cardiovascular risk factors, the opiate dose-duration interaction was significant and appeared in 12 terms in the final model. It was also independently significant (P = 0.0153). Opiate dose or duration appeared in a further 15 terms. The model was shown to be significantly improved by the inclusion of terms for opiate dependency (A.I.C. 71.10 v 54.31, P < 0.0001).

**Conclusion:**

These data confirm increased vascular stiffness and ageing in a longitudinal study, and thereby imply advanced organismal ageing. These multivariate studies are consistent with opiate dependency as an interactive and multivariate cardiovascular risk factor and emphasize the role of treatment duration.

## Introduction

Up to 90 million Americans report chronic pain which is increasingly managed by use of oral opiates at an annual cost of over $100 billion [1]. The National Comorbidity Survey in 1994 showed that over 7% of Americans reported a lifetime history of drug dependence and 3% of adult Americans reported a current dependence. Associated with prescription opiate treatment is an increase in opiate overdoses which now outstrips combined overdoses associated with illicit heroin or cocaine. Between 1998 and 2009 USA emergency room (ER) overdose rates from non-methadone non-heroin opiates increased from 2.2% to 11.5% [2].

Several studies have shown an association between opiate dependence and high rates of cardiovascular disease [3, 4], with data indicating increased rates of hypertension, heart disease and diabetes at 52.2%, 18.5% and 16.3% in methadone patients age over fifty [4]. A large Australian post mortem study found that 17% of a group of heroin users 44 years of age and older had a coronary artery stenosis of 75% or more [5]. Large post mortem based studies in the USA [3] and Australia [6] give results in close agreement in terms of the excess mortality rate observed in opiate users. In a 33-year follow-up study in the U.S. the average number of years of life lost to cardiovascular disease in the census population of both White and Hispanic populations was 8.3/1,000 in 1997 , whilst in drug dependent patients it was 26.6 and 31.2/1,000 respectively [3]. An Australian study involving a 20 year follow-up of over 42,000 patients (representing greater than 430,000 patient-years) receiving opiate pharmacotherapy for management of heroin dependence found a standardized mortality rate of 2.2 (95%C.I. 1.8 - 2.5) for death by cardiovascular disease [6]. Asian studies found very similar results with one group reporting an odds ratio of 1.8 (C.I. 1.1 - 3.1; P = 0.001), persistence of the relationship when the analysis was limited only to non-smokers, a dose response relationship between lifetime dose of opiates consumed and the severity of coronary disease, and an earlier occurrence of coronary disease requiring surgical revascularization in the opiate exposed group four years before that of the comparator surgical group [7, 8]. Indeed in Iran opium consumption is one of the most important coronary risk factors [9]. Moreover the Australian group has further shown an overall adjusted odds ratio for cardiac pathology in methadone compared to non-methadone decadents of 3.13 (C.I. 2.00 - 4.90) which may be related to the higher degree of opiate agonism achieved in the latter group [10]. Nevertheless prospective antemortem studies of this problem, which is presumably becoming of increasing importance as the frequency of opiate use in the community rises, are lacking.

Atherosclerosis and coronary artery disease in particular are often said to be complex polygenic disorders of multifactorial causality. Hence several genome wide association studies (GWAS) which reported a consistent association of coronary disease with a “gene desert” on the short arm of chromosome 9p21.3 in the senescence locus, caused much interest [11]. The site is near the major senescence and cancer associated genes CDKN2A and CDKN2B coding for P16INK4A, P15INK4B and P19ARF, but actually maps to a non-protein coding segment coding for the long RNA called ANRIL, which has since been shown to interact in cis, via γ-interferon and possibly via common sense and anti-sense activation of their shared promoter region, with P16INK4A [12]. It is therefore of great interest that the growth inhibitory activities of opiates which have been described and studied for many years [13], have recently been shown to be related to their activities with P16INK4A [14], which is mediated by a special cloned and sequenced perinuclear receptor known as the opiate growth factor receptor [15]. It is also important to note that the growth inducing and senescence promoting effects of opiates can all be reversed from -30% to + 30% pharmacologically by opiate antagonists [15] which are sometimes used to treat clinical opiate dependence. Hence opiate dependence theoretically forms a complex but important model for contributing to our understanding of atherosclerotic processes, which is consistent with the above cited epidemiological studies.

For several reasons such studies in opiate dependent patients are challenging. The group may have non-ideal compliance with appointments and schedules, and is notoriously difficult to track in the long term. More particularly the mean age in most series is usually early in the fourth decade, when many tests of vascular function have limited ability to detect important preclinical changes. Pulse wave analysis (PWA) by radial arterial tonometry overcomes this methodological hurdle. It is based on the observation that the central pressure waveform experienced by the large arteries, aortic root and therefore the left ventricular outflow tract, is very different to that measured over the brachial artery. The central pressure waveform may be deconvoluted into a forward moving incident pressure wave arising from ventricular activity and the reflected waveform returning from peripheral resistance sites. The speed and amplitude of this reflected waveform is related to the stiffness of the arterial system, and so the vascular age. As the backward wave augments the pressure of the forward wave related in part to the timing of its arrival in the central vessels, this additional pressure is known as the augmentation pressure. It may be calibrated for the pulse height, when it is known as an augmentation index. Both are calibrated in absolute terms by the brachial pressure, which allows back-calculation of the central pressures by a validated transfer function.

Since this clinic sees both opiate dependent and general medical patients and has experience with the technique of Pulse Wave Analysis (PWA), it seemed that this constituted at once an unusual and ideal opportunity in which to investigate and quantify the effects of long term opiate agonist treatment on the important subclinical endophenotypes of central arterial stiffness and vascular age both antemortem and longitudinally. Moreover this work relates specially to females which with few exceptions [16], have been have been largely overlooked in the extant literature.

## Methods

### Patient selection and treatment

Thirty seven female control patients aged 38.72 (SE 2.64) years were recruited opportunistically from patients presenting for health examination checks for insurance or employment medical examinations or from patients presenting with minor health problems. Ninety three opiate dependent patients treated currently under treatment with buprenorphine (n = 84: 90.3%), methadone (n = 4: 4.03%) or naltrexone implant (n = 5: 5.4%) were recruited and sampled opportunistically at the time of their clinic visits. Opiate dependent patients were treated by accepted clinical algorithms by their usual health providers for their drug dependency. The mean daily doses of buprenorphine and of methadone which were used were 7.05 ± 0.58mg, and 46.25 ± 8.98 mg respectively. Further details of drug use in this cohort have been previously reported [17, 18]. The naltrexone implants used for the management of heroin dependence were manufactured by “Go Medical” industries in Perth, Western Australia under Commonwealth of Australia Therapeutic Goods Administration (TGA) GMP conditions and administered under the compassionate Access arrangements of the Special Access Scheme authorized by the TGA. This treatment has been described in detail elsewhere [18]. Patients were recalled for retesting at two and five years post baseline assessment. Any patient with a known chronic or subacute cardiovascular condition such as hypertension, stroke, coronary artery disease, diabetes, renal failure, pregnancy or infectious disorder was excluded from participation. Frequency and quantity of substance use by substance type (inclusive heroin) was assessed at time of initial and retesting. Other opiates were converted into morphine equivalents, and then into heroin equivalents at the rate of 1g of street heroin = 0.5 g morphine. The duration of opiate use was counted from the time of first use.

### PWA studies

Patients were positioned supine for the performance of the test and allowed to rest for five minutes prior to the test. Consumption of food, drink, alcohol or tobacco was not restricted, but patients were not allowed either to sleep or talk during the testing procedure. If it became known that alcohol had been consumed in the few hours prior to the test, the studies for that day were excluded. The Miller microtonometer was positioned over the right radial artery unless it was unavailable. The brachial blood pressure was taken in the opposite arm using the oscillometric Omron HEM 907 device. The data was collected and analyzed by the SphygmoCor software. Patients were studied in quintuplicate, and adequate studies were averaged for that day. Acceptable studies had an Operator index of greater than 70%, and were not inconclusive on initial reporting by the software. Details of patients prior drug use was also taken at the time of the study performance and entered into the database. Time in days was measured from the time of the first PWA study.

The SphygmoCor software used for the PWA studies calculates a number of major indices including the Vascular or Reference Age (VA, RA), the Chronologic Age (CA), the Central Augmentation Pressure at Heart Rate 75 (C-AP-HR75) the Central Augmentation Pressure/Pulse Height Ratio at Heart Rate 75 (C-AGPH-HR75) also known as the Augmentation Index, Central Pulse Height (C-PH), Peripheral - Central Pulse Pressure Amplification Ratio (PPAmpRatio), Central Systolic Pressure (C-SP), Central Diastolic Pressure (C-DP), Central End Systolic Pressure (C-ESP), Central Mean Pressure (C-MEANP), the Central Diastolic Time Index (C-DTI), the Central Tension Time index (C-TTI), the Central Diastolic Duration (C-DD), and an index of subendocardial perfusion known variously as the Subendocardial Perfusion Ratio (SEVR), the Central Stroke Volume Index (C-SVI) or the Buckberg ratio, which is defined as the C-TTI/C-DTI.

### Statistics

Data are presented as the mean ± S.E.M. Categorical data were studied using “EpiInfo” 7.0.8.3 obtained from the CDC Atlanta, Georgia using the Chi squared test, or the Fisher Exact test if the numbers in a cell were less than 20. Continuous data were analyzed in “Statistica” from Statsoft Oklahoma using Student’s t-tests. T-tests with separate variances were used where Levene’s test was significant. Continuous variables such as HDL, BMI, and systolic pressure were log transformed in accordance with normality assumptions. CRP and the heroin dose were arcsinh transformed which is a similar transform to logarithmic, but accepts arguments of zero. Time (as days) was log transformed as described. The time since the last cigarette was studied as a factor with cut-off points at 30, 60, 120, 180 and > 180 minutes, and a non-smoking category. Time dependent repeated measures analysis was performed using non-linear mixed effects regression module in “R” 2.13.1 obtained from the Comprehensive “R” Archive Network mirror at the University of Melbourne. Model reduction was performed by visual inspection and removal of the least significant terms. Only significant terms remained in the final models which are presented. Restricted equation maximum likelihood (REML) techniques were used by default unless otherwise specified. Random effects were assigned to a patient identification code and arcsinh time, unless otherwise stated. Ggplot2 software was used to prepare graphs. P < 0.05 was considered significant.

### Ethical approval

All patients provided their informed consent to participate in the study and for their medical treatment. The study was approved by the Human Research Committee of the Southcity Family Medical Centre, which has been registered with the National Health and Medical Research Council.

## Results

A total of 37 control females and 93 opiate dependent females were studied on each of 117 and 275 occasions (2.71 ± 0.19 and 2.35 ± 0.093 studies each, mean ± S.E.M., log data P = 0.26). As shown in Table 1 the mean ages were 38.72 ± 2.64 and 33.78 ± 0.90 (log data P = 0.032), 10.81% and 91.40% smoked tobacco (Fisher Exact P < 0.0001). Significant differences were noted in the body mass index (BMI) in the two groups, and for tobacco and opiate consumption. ESR and serum globulins were also higher in the opiate exposed group as has been reported [19]. Of the opiate dependent patients, 84 (90.3%) were treated with buprenorphine, 5 (5.4%) were treated with naltrexone, and 4 (4.03%) were treated with methadone.

**Table 1:** Sociodemographic and Laboratory Data

Parameter	Control	Opiates	P
No.	37	93	
No. of Studies*	117	182	0.7621
Chronologic Age	38.72 (2.64)	33.78 (0.9)	0.0260
Biometrics			
Height (cm)	163.59 (1.13)	165.66 (0.64)	0.0995
Weight (kg)	67.05 (2.39)	62.56 (1.2)	0.0988
Body Mass Index (kg*m^-2^)	25.09 (0.91)	22.76 (0.39)	0.0224
Tobacco			
Cigarettes/Day	1.89 (1.08)	16.05 (0.98)	0.0000
Smoking-PWA-Interval (Min.)	97.19 (2.7)	51 (4.83)	0.0000
Heroin			
Heroin Duration (Years)	0 (0)	12.33 (0.86)	0.0000
Heroin Dose (g)	0 (0)	0.45 (0.04)	0.0000
Heroin-Dose-Duration(g-Yrs)	0 (0)	5.28 (0.52)	0.0000
Lab Values			
Cholesterol (mmol/L)	4.8 (0.19)	4.73 (0.11)	0.7697
HDL (mmol/L)	1.56 (0.11)	1.51 (0.05)	0.6797
LDL (mmol/L)	2.63 (0.17)	2.56 (0.12)	0.7855
Triglycerides (g/L)	1.17 (0.18)	1.1 (0.06)	0.6457
CRP* (g/L)	1.48 (0.33)	6.4 (2.78)	0.1826
ESR (mm/hr)	9.875 (1.53)	14.72 (1.9)	0.0498
Globulins (g/L)	29.54 (0.83)	32.63 (0.59)	0.0083
Platelets (× 10^9^/L)	305.33 (16.31)	268.67 (8.54)	0.0488
Neutrophils(× 10^9^/L)	10.51 (3.62)	8.68 (1.49)	0.5899

*: Statistics for Log Parameters Reported.

The period of study was 0 - 1,797 days (mean 364.58 ± 23.87). The controls were studied for 368.85 ± 43.83 and the opiate dependent patients were studied for 362.69 ± 28.50 days (t = 0.407, df = 118, P = 0.68).

Various cardiovascular parameters are shown in Table 2. No significant differences were demonstrated.

**Table 2:** Cardiovascular Data

Parameter	Control	Opiates	P
Operator Index	88.03 (1.11)	87.51 (0.68)	0.6848
Age Indices			
RA*	43.65 (3.76)	37.29 (1.87)	0.2451
RA-CA-Difference	4.87 (2.59)	3.47 (1.46)	0.6233
RA/CA*	1.12 (0.06)	1.09 (0.04)	0.5682
Augmentation Indices			
C-AP-HR75	6.27 (1.16)	5.55 (0.51)	0.5730
C-AGPH-HR75	15.27 (2.87)	15.03 (1.27)	0.9399
C-PH	35.11 (1.69)	34.92 (0.78)	0.7672
PPAmpRatio	144.86 (4.13)	144.82 (2.07)	0.9918
P-AI	69.05 (4)	68.69 (1.9)	0.9345
Pressure Indices			
SP*	119.81 (2.68)	116.34 (1.06)	0.3169
DP*	70.49 (1.87)	66.71 (0.96)	0.0600
C-SP*	107.03 (2.85)	102.99 (1.13)	0.2796
C-DP*	71.92 (1.88)	68.13 (0.97)	0.0620
C-MEANP	88.05 (2.17)	84.38 (0.96)	0.0737
C-ESP	95.51 (2.6)	91.89 (1.1)	0.1323
Timing Indices			
HR	69.86 (1.76)	70.14 (1.09)	0.8940
ED	330.57 (3.04)	329.15 (2.06)	0.7086
C-SVI	140.16 (5.08)	138.52 (2.7)	0.3842
C-TTI	2242.32 (75.85)	2151.28 (37.3)	0.2862
C-DTI	3035.46 (85.87)	2905.08 (37.86)	0.1088
C-DD	550.73 (20.22)	548.38 (12.83)	0.9221

*: Statistics for Log Parameters Reported.

**Figure 1:**
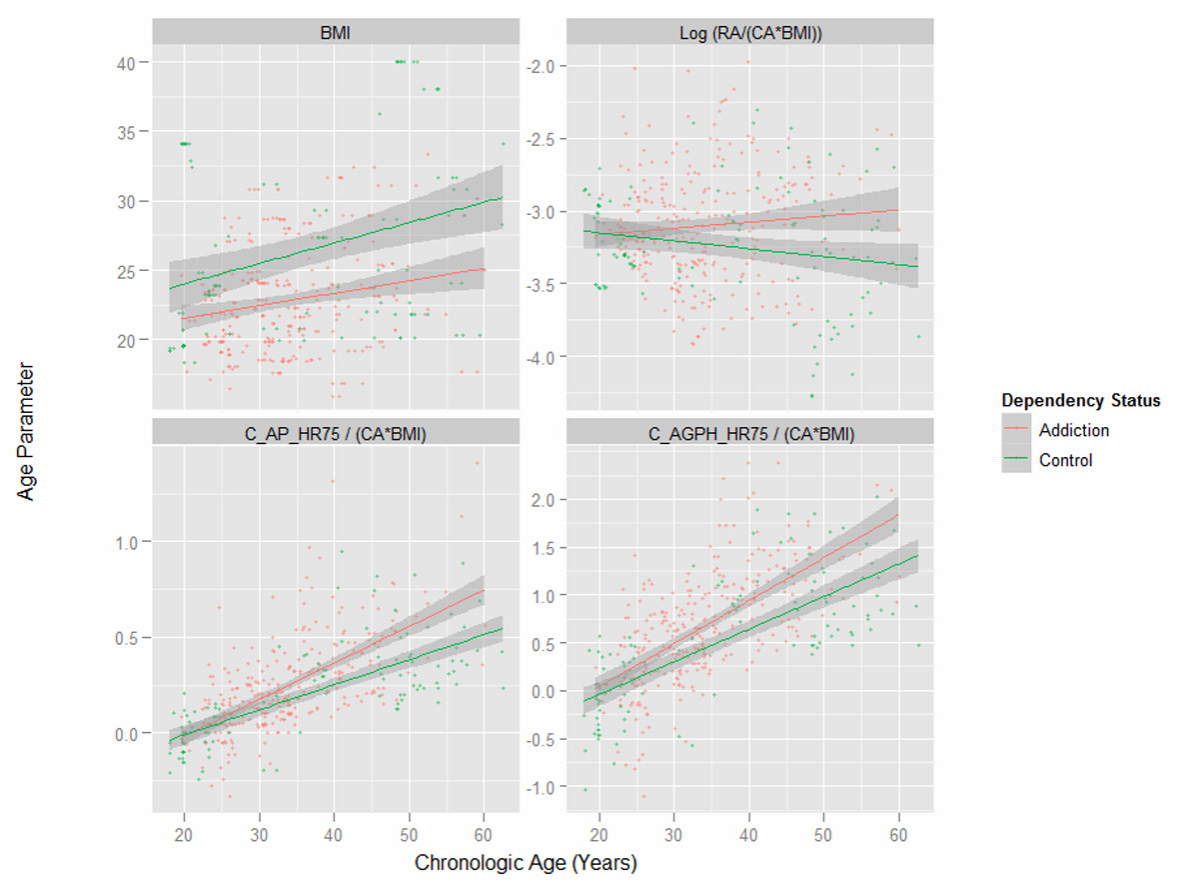
Corrected ageing and stiffness indices by chronologic age by opiate dependency status.

**Figure 2:**
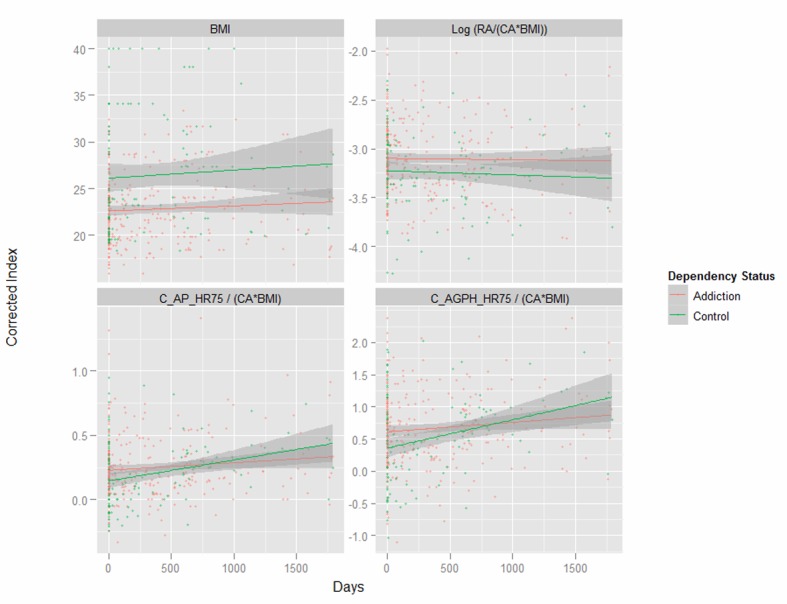
Corrected ageing and stiffness indices by time age by opiate dependency status.

**Figure 3:**
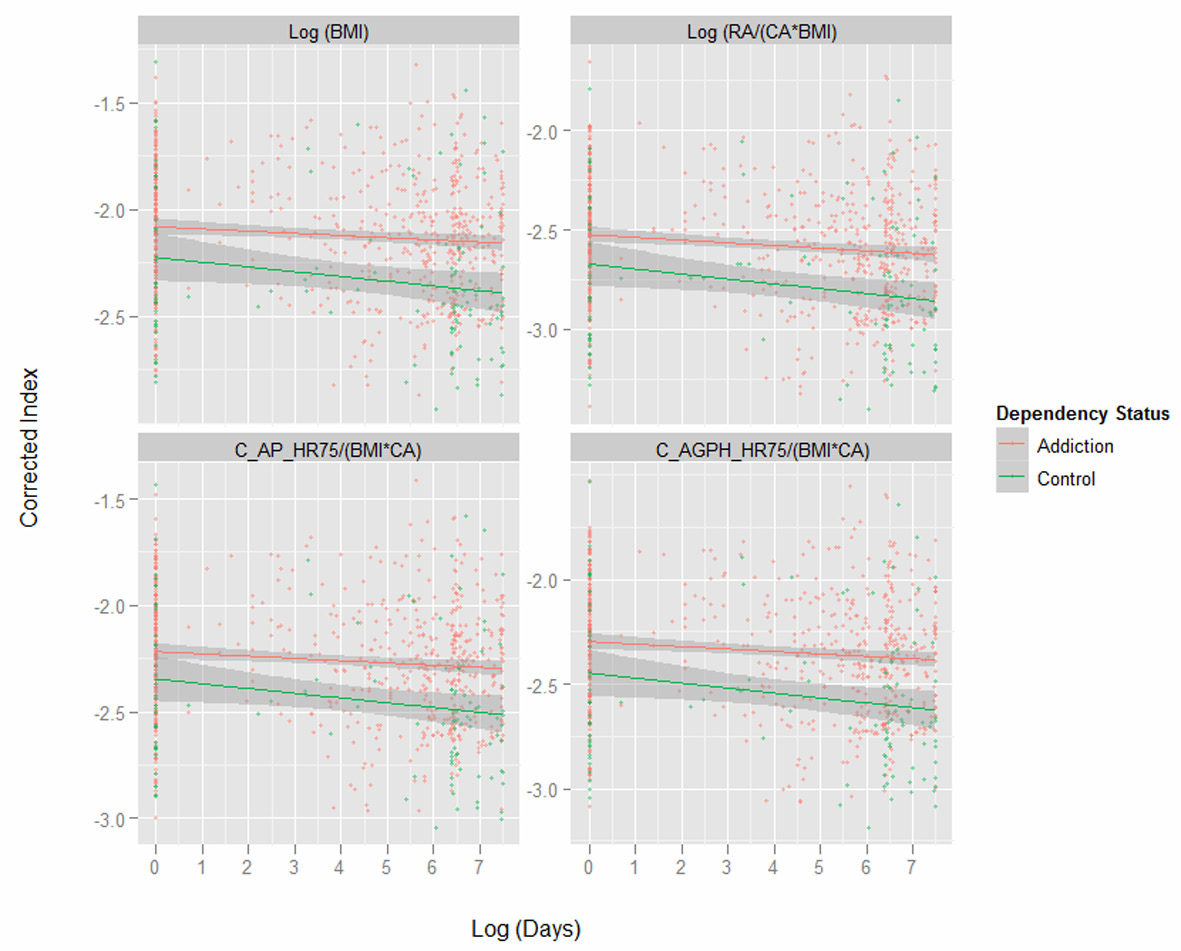
Corrected indices by Log (time) age by opiate dependency status.

**Figure 4:**
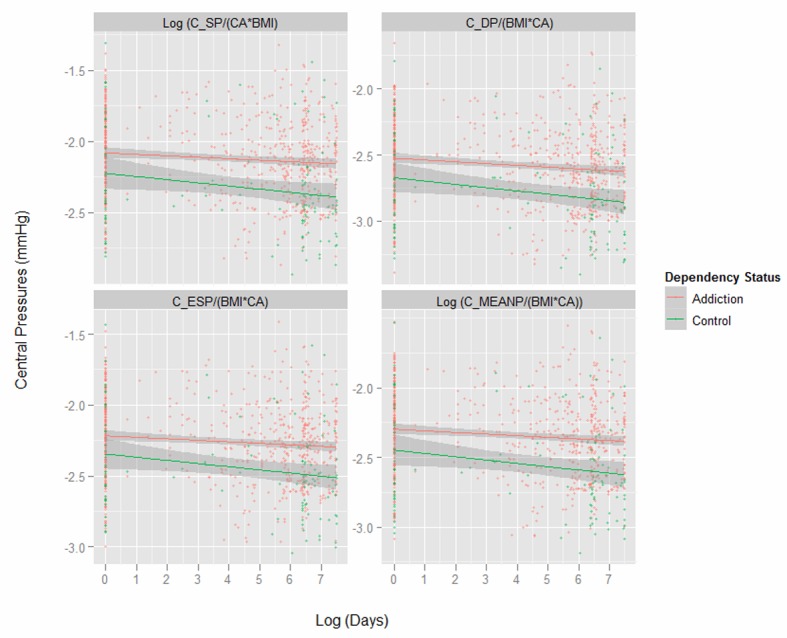
Central pressures by chronologic age by opiate dependency status.

Figure 1 presents key variables by patient chronological age. One notes the dramatic difference in the body mass index (BMI) between the two groups shown in the first panel. Subsequent indices are therefore adjusted for both age and BMI as indicated. Figure 2 shows the same variables by time elapsed on the study. Figure 3 shows the same variables by arcsinh time under observation. Figure 4 shows some key pressure variables by arcsinh time, and notes a clear separation between the groups.

There were baseline differences in the BMI between the two groups (Fig. 1). When this was analyzed formally and the log of the BMI was regressed (log) time and addictive status with unity and the patient codes as random covariates, the addictive status was significant both in its own right (est. = 0.000967 dF = 260, P = 0.0185) and in interaction with time (est. = 0.00289, dF = 260, P = 0.0025; model AIC = -884.002, log likelihood ratio = 448.001).

Table 3 presents a summary of key central cardiovascular variables regressed against (log) time, (log) BMI and opiate dependency status. The random effects in each case were the patient code and (log) time, except as indicated. Many of these variables are noted to vary significantly by dependency status alone, or in interaction with time or BMI.

**Table 3:** Central Cardiovascular Measures Standardized Against Age and BMI

Parameter	Variable	Value	Std. Error	DF	t-value	P-value	AIC	Log Lik
RA	Opiate. Status	-1.1387	0.5654	159	-2.0138	0.0457	254.30	-119.15
RA	BMI: Opiate. Status	0.3590	0.1771	159	2.0269	0.0443	254.30	-119.15
C-AGPH-HR75*	Opiate. Status	0.1643	0.0637	260	2.5797	0.0104	178.44	-84.22
C-AP-HR75	Opiate. Status	0.0400	0.0148	161	2.7138	0.0074	-422.44	218.22
C-SVI	Opiate. Status	-1.3100	0.6112	159	-2.1435	0.0336	323.03	-152.52
C-SVI	BMI: Opiate. Status	0.3931	0.1915	159	2.0528	0.0417	323.03	-152.52
C-SP*	Opiate. Status	-1.5297	0.6029	259	-2.5371	0.0118	-450.39	232.19
C-SP*	BMI: Opiate. Status	0.4994	0.1902	259	2.6248	0.0092	-450.39	232.19
C-DP	Opiate. Status	-2.0041	0.7374	259	-2.7177	0.0070	-262.00	138.00
C-DP	BMI: Opiate. Status	0.6435	0.2325	259	2.7679	0.0060	-262.00	138.00

All Parameters divided by CA; All parameters log transformed except C-AP-HR75 and C-AGPH-HR75; Days refers to log (Days); Opiate Status refers to drug dependent status; Dependent variable in Column 1. Regressed against interactions between log (CA), log (BMI) and dependency status; *: For technical reasons random effects for these variables were unity and patient code.

Table 4 presents a final model after classical manual model reduction of an exhaustive repeated measures multivariate model which includes interactive terms in time, opiate dose and duration, tobacco consumption, BMI, and CRP, and additive terms in time since last cigarette, HDL, brachial systolic pressure, cholesterol and height. Random effects were unity and the patient identification code. The model parameters included an AIC of 54.3066, log likelihood ratio = 18.8466. Of the 40 terms in the final model 12 include the opiate dose-duration interaction, and this interaction also occurs alone (est. = -1.99, P = 0.0153). Opiate dose or duration occur a further 15 times in the model.

**Table 4:** Final Multivariate Model for Log (RA/CA)

Parameter	Value	Std. Error	DF	t-value	P-value
SP	1.5958	0.2782	77	5.7353	0.0000
Days: H. Dose: H. Dura. n: CRP	-0.3689	0.0834	77	-4.4213	0.0000
Days: H. Dose: H. Dura. n: Cigarettes: CRP	0.0149	0.0034	77	4.3597	0.0000
Days: H. Dose: H. Dura. n: BMI: CRP	0.1205	0.0274	77	4.3977	0.0000
Days: H. Dose: H. Dura. n: Cigarettes: BMI: CRP	-0.0049	0.0011	77	-4.3208	0.0000
Days: Cigarettes	0.1847	0.0434	77	4.2569	0.0001
Days: Cigarettes: BMI	-0.0579	0.0138	77	-4.2058	0.0001
Days: Cigarettes: CRP	-0.0498	0.0122	77	-4.0976	0.0001
Days: Cigarettes: BMI: CRP	0.0162	0.0039	77	4.1599	0.0001
Days	-2.8302	0.7264	77	-3.8964	0.0002
H. Dose: H. Dura. n: Cigarettes: CRP	-0.0876	0.0228	77	-3.8454	0.0002
H. Dose: H. Dura. n: Cigarettes: BMI: CRP	0.0297	0.0076	77	3.9090	0.0002
Days: H. Dura. n	0.1886	0.0500	77	3.7690	0.0003
Days: BMI	0.8776	0.2298	77	3.8191	0.0003
Days: H. Dura. n: BMI	-0.0589	0.0157	77	-3.7608	0.0003
Days: H. Dose: CRP	2.5007	0.6759	77	3.6997	0.0004
Days: H. Dose: BMI: CRP	-0.8054	0.2173	77	-3.7070	0.0004
Minutes. Since. Cigarette = 120	2.0219	0.5760	77	3.5101	0.0008
Height	-0.0182	0.0056	77	-3.2292	0.0018
H. Dose: H. Dura. n: Cigarettes: BMI	-0.0440	0.0139	77	-3.1570	0.0023
H. Dose: H. Dura. n: Cigarettes	0.1305	0.0422	77	3.0947	0.0027
Cigarettes: BMI	-0.0080	0.0027	77	-3.0058	0.0036
H. Dose: H. Dura. n: BMI: CRP	-0.4659	0.1562	77	-2.9818	0.0038
Days: H. Dura. n: Cigarettes	-0.0079	0.0027	77	-2.9509	0.0042
Days: H. Dura. n: Cigarettes: BMI	0.0024	0.0008	77	2.9358	0.0044
H. Dose: H. Dura. n: CRP	1.3888	0.4748	77	2.9248	0.0045
Days: H. Dose: Cigarettes	-0.2159	0.0829	77	-2.6031	0.0111
HDL	-0.3307	0.1266	62	-2.6127	0.0113
H. Dose: Cigarettes: BMI	0.2675	0.1037	77	2.5808	0.0118
Days: H. Dose: Cigarettes: BMI	0.0697	0.0273	77	2.5496	0.0128
H. Dose: H. Dura. n: BMI	0.6719	0.2645	77	2.5407	0.0131
H. Dose: Cigarettes: BMI: CRP	-0.1300	0.0514	77	-2.5294	0.0135
H. Dose: Cigarettes: CRP	0.3834	0.1542	77	2.4867	0.0151
H. Dose: H. Dura. n	-1.9913	0.8027	77	-2.4808	0.0153
H. Dose: Cigarettes	-0.7700	0.3141	77	-2.4517	0.0165
Minutes. Since. Cigarette = Non-Smoker	-0.3387	0.1431	77	-2.3665	0.0205
H. Dose: BMI	-0.3108	0.1350	77	-2.3020	0.0240
Days: H. Dose	3.4102	1.5421	77	2.2114	0.0300
Days: H. Dose: BMI	-1.0954	0.5098	77	-2.1487	0.0348
Minutes. Since. Cigarette = 180	0.7860	0.3825	77	2.0550	0.0433

This model was compared with an identical model which replaced the linear term in opiate duration with a squared term. The AIC of the final model was 53.7227 and the log likelihood ratio 20.1441. The ANOVA comparison with the linear model was not significant (log ratio = 2.59, P = 0.1072).

This model was further compared with a model which had no terms for opiate exposure in it, but was otherwise identical to the linear model just described. The AIC was 71.10 and the log-likelihood ratio was -13.55. At ANOVA testing the log ratio for comparison of these two models was 64.7958, P < 0.0001. This demonstrates that inclusion of terms for opiate exposure in the model significantly improves it.

## Discussion

The principal findings of this study were that when corrected for age and body mass index opiates appeared to have a profound and deleterious effect on central cardiovascular parameters particularly indices of arterial stiffness, pressure and vascular ageing, and that they correlated strongly and persistency with CA corrected vascular age in a fully adjusted model, with effects appearing both independently and through multiple interactions with established risk factors. The lifetime opiate dose-duration exposure was shown to be significantly (P = 0.0153) and independently related to the (log) RA/CA ratio demonstrating a direct dose-response effect. It also featured in a further 11 terms in the final multivariate model, showing that the lifetime opiate exposure is related both independently and interactively to indices of vascular stiffness. The opiate dose and duration were separately featured in a further 15 terms in the final model, so that they feature together or alone in 27 of the forty terms remaining in the final model. Indeed a model accounting for the rise in vascular age with time including all known risk factors was significantly improved by the inclusion of terms relating to lifetime opiate use (P < 0.0001). Hence these observational findings from this multivariate study offer important corroborating evidence to strengthen the findings quoted in the Introduction relating to the probable role of opiate abuse in the development of cardiovascular disease.

A further corollary of these findings from the literature is that, with the central importance of the vasculature to human health, vascular ageing is a key biomarker for ageing of the system generally. Published results from large surveys and trials [3-8] as well as our previously published results from this clinic [17, 19, 20] are consistent with the view that the health of opiate dependent patients suffers a long term decline which is generalized across all body systems examined, and is consistent with an accelerated ageing effect.

It has also been noted that as over half the population of western nations will die from cardiovascular disease, more than half the effect of ageing can be ascribed to cardiovascular ageing [21]. For this reason the importance of the present studies extends well beyond cardiovascular medicine. For example the demonstration that opiate dependent patients’ vasculature was ageing in a more accelerated manner would be consistent with other studies showing this in hair greying, dental disease, immune profile, hyperglycaemia, and osteoporosis [17, 19, 20, 22-24], and would therefore carry major implications for the period for which opiate agonist treatment might be safely recommended, which at present, is indefinite [4-6, 10, 25-27].

This study also documented a very concerning trend to increased weight and body mass over time in the control group in our cohort, which reflects a common problem in the Australian community at this time. It is noted that the opiate dependent patients did not appear to be as susceptible to this trend as the control group (P = 0.0185 for its effect as a factor, and P = 0.0025 for its interaction with time). Interestingly this is contrary to the strong evidence of the published literature in terms of the stimulation by opiates of the appetitive drives for high fat, high carbohydrate foods [23, 28, 29]. It is possible that it most likely reflects the diversion of resources away from self-care into drug use, in terms of the finite reach of limited resources associated with the drug dependent lifestyle and the frequent diversion of funds.

As mentioned earlier these findings provide a basis for examining patients for signs of senescence and immune activation in the cardiovascular system and more generally. Study findings are consistent with previously published work documenting high rates of cardiovascular disease in opiate dependent patients in America, Australia and the Middle East [3, 5-8]. They are also consistent with evidence from other multiple organ systems showing signs of advanced ageing in opiate users [4, 5, 17, 19, 20]. Accordingly opiate dependent persons might prove a useful model to study for signs of senescence and immune activation and their long term sequelae associated with opiate agonist and antagonist activity.

This study also buttresses other recently published work which suggests increased arterial ageing in opiate dependent patients in a cross-sectional study (manuscripts in press). Although in this study when the linear model was compared to the model with the quadratic term in opiate duration no significant difference was found, the smaller sample size, and the broadly similar effect to that found in males suggest that the effect may yet exist as sample accrual continues, or if larger studies or different investigative techniques are performed.

Findings both from this clinic and in the literature suggest that protracted use of full opiate agonists are accompanied by severe arteriopathic changes after the longer term [10]. That only 4% of the present sample were treated with the full opiate agonist methadone, and the mean dose of buprenorphine, a partial agonist, was low by published standards, the present study likely represents a best case scenario for the estimation of the long term arteriopathic effects of opiate agonism, and as such a lower bound for the likely effect of full opiate agonists. That arteriopathic changes appear to continue to increase during long term opiate exposure is of great concern since the golden standard for management of heroin dependence is long term opiate, with patients caught in a compounding pathophysiologically interest situation. Current findings clearly bring into question the current practice of indefinite opiate agonist or partial agonist maintenance for many classes of opiate dependent persons [5, 6, 27, 30], and highlight the need for cost benefit assessment and the need for non-opiate agonist/partial agonist long term therapeutic options.

Finding that both the CA and BMI corrected central systolic and diastolic pressures were elevated is of prognostic significance given the known association of indicators with all cause and cardiovascular mortality [31, 32]. Similarly the heart rate corrected central augmentation pressure and central augmentation index were elevated in these patients which has also been linked to mortality and cardiovascular outcomes [33].

Many coronary risk factors have been shown to be elevated in opiate dependent patients, which are likely related to their pharmacological effects. Hence studies link opiates and weight gain, hyperglycaemia and diabetes, hypertension, dyslipidaemia and increased tobacco usage [34]. This has implications for studies including the present one which seek to use multivariate methods to define a role for opiate dependence in various target disorders. Such models would actually be overcorrecting for the opiate related effects. To adjust for CRP level is not methodologically valid when significant immune perturbations have been noted repeatedly and classically [27] in opiate treated cultured cells, animal models and patients. Similar comments apply to hypertension and dyslipidaemia.

The co-occurrence of a metabolic syndrome like profile [34], immunostimulation, immunosenescence and immunosuppression is noteworthy and has been remarked upon in other situations as being a singularly adverse combination [35]. Such factors are believed to act independently and interactively, much as was demonstrated in this study between CRP, HDL and brachial systolic pressure. In the case of the present work opiates were shown at multiple regression to interact with both these groups of factors. The above discussion shows that these statistical findings likely have a mechanistic underpinning, and point to a fundamental understanding of opiate induced pathophysiology and accelerated age related decline.

The interplay of chronic inflammation in our patients systemically, and presumably in their vessel walls as is now realized in atherosclerotic disease [36], and the cellular senescence pathways described above is also fascinating [11, 14]. Many interactions have been shown between pro-inflammatory and pro-senescent pathways. Clearly it implies that cells are less fit to cope with inflammatory insults. Senescent cells have now been characterized as secreting many proinflammatory cytokines including interleukins -6 and -8, which potentiate and maintain the senescent state. And inflammation is a powerful inhibitor of regenerative activities in most stem cell niches.

The ability of opiates to exacerbate chronic inflammatory disorders has been noted both by ourselves [20, 24] and others [7, 8]. Similar findings have been made in relation to laryngeal cancer [37], and it is likely that this interactive and potentiating effect plays a role in other published series of cancer and addiction [3, 6, 38]. As links are increasingly being demonstrated between chronic inflammatory states and cancer, and between chronic inflammation and ageing it would seem that more conceptual cross-fertilization would follow as more mechanistic interplay is demonstrated at the molecular level.

This study has several limitations. Some of the most important of these are related to the observational design of this study. As such it cannot be said to represent a definitive study of the central hypothesis. The data however is highly suggestive and entirely consistent with a published body of research from several continents. It is therefore felt that a prospective trial examine the health of variously treated opiate dependent patients and matched control groups is indicated to examine these issues in more detail. Naturally it is accepted that randomization to an opiate dependent condition is not ethically permissible, but randomization within the opiate dependent cohort could be arranged, and careful age and sex matching of a control group could also be organized from various population databases.

Only 4.3% of the opiate dependent patients in this study were treated with the full opiate agonist methadone, with the remainder of patients treated with a relatively low dose of the partial opiate agonist buprenorphine. There is evidence that full opiate agonists such as methadone may have a different and more deleterious profile of cardiovascular disorders than others [10]. Hence the results presented here may actually constitute a best case scenario for opiate induced vascular toxicity. Thus future studies should take care to recruit patients treated with a wider variety of opiate agonist, partial agonist, and antagonist agents, delivered at different levels. The narrative form of the drug history taken in these patients was not conducive to formal analysis and care should be taken to address this in the future. Other methods of cardiovascular assessment were not available to this work, with the case for an association between opiate use and increased cardiovascular disease strengthened by use of other methods of cardiovascular assessment. Similarly tests for ageing, senescence and other tests in other tissues could be applied to such cohorts in future replications of this type of research.

The present study has raised many intriguing questions in the relationship between opiate receptor stimulation and subclinical arterial stiffness and cardiovascular disease. It is hoped that subsequent work powered to simultaneously investigate the numerous mechanistic leads suggested by these results will advance the conceptual understanding of opiate related vasculopathy in these patients and also of atherogenesis and vascular ageing in the wider community.
